# Supplementary material for: Multiplex PCR Detection of Enteric Pathogens in a Community-based Birth Cohort in Ecuador: Comparison of xTAG-GPP and TaqMan Array Card Assays
Source: Open Forum Infect Dis. 2025 Jan 17;12(2):ofaf027. doi: 10.1093/ofid/ofaf027 (PMC11800474; doi:10.1093/ofid/ofaf027)

Supplementary Information File

Multiplex PCR detection of enteric pathogens in a community-based birth cohort in Ecuador: comparison of xTAG-GPP and TaqMan array card assays

Stuart Torres Ayala, Lesly Simbaña Vivanco, Nikolina Walas, Kelsey Jesser, Nicolette A. Zhou, Christine S. Fagnant-Sperati, Hadley R. Burroughs, Gwenyth O. Lee, Joseph N.S. Eisenberg, Gabriel Trueba, Karen Levy, Benjamin F. Arnold

Open Forum Infectious Diseases

Contents

Supplemental Table 1
Luminex Gastrointestinal Pathogen Panel (GPP) assay targets and corresponding median fluorescence intensity (MFI) thresholds for positivity

Supplemental Table 2
TaqMan Array Card (TAC) assay gene targets and corresponding forward (F) and reverse (R) primers and probe (P) sequences.

Supplemental Table 3
Infection prevalence for 14 enteric pathogen targets measured by Luminex xTAG Gastrointestinal Panel (GPP) and TaqMan Array Card (TAC) assays.

Supplemental Figure 1
Median fluorescence intensity (MFI) values for pathogen targets detected by the Luminex GPP Assay.

Supplemental Figure 2
Cycle threshold (Ct) values for pathogen associated gene targets detected by the TAC Assay.

Supplemental Figure 3
Number of pathogens detected in each sample by assay.

Supplemental Table 1: Luminex Gastrointestinal Pathogen Panel (GPP) assay targets and corresponding median fluorescence intensity (MFI) thresholds for positivity

**Analyte**	**Threshold for positivity (MFI)**
Adenovirus *40/41*	≥ 150
Camplobacter	≥ 150
C. difficile Probe-1	≥ 150
C. difficile Probe-2	≥ 150
Cryptosporidium	≥ 250
E. coli O157	≥ 150
E. histolytica	≥ 250
ETEC probe-1	≥ 200
ETEC probe-2	≥ 200
Giardia	≥ 250
Norovirus Probe-1	≥ 200
Norovirus Probe-2	≥ 350
Rotavirus A	≥ 150
Salmonella Probe-1	≥ 100,000 (POS), < 300 (NEG)
Salmonella Probe-2	≥ 200
STEC Probe-1	≥ 150
STEC Probe-2	≥ 150
Shigella	≥ 150
V. cholerae	≥ 150

Supplemental Table 2: TaqMan Array Card (TAC) assay gene targets and corresponding forward (F) and reverse (R) primers and probe (P) sequences.

Organism	Gene Target	Sequence
Adenovirus (40/41)	*Fiber gene*	*F, AACTTTCTCTCTTAATAGACGCC*<br>*R, AGGGGGCTAGAAAACAAAA*<br>*P, FAM-CTGACACGGGCACTCT-MGB*
Norovirus GI	*ORF1-ORF2*	*F, CGYTGGATGCGNTTYCATGA*<br>*R, CTTAGACGCCATCATCATTYAC*<br>*P, FAM-TGGACAGGAGATCGC-MGB*
Norovirus GII	*ORF1-ORF2*	*F, CARGARBCNATGTTYAGR TGGATGAG*<br>*R, TCGACGCCATCTTCATTCACA*<br>*P, FAM-TGGGAGGGCGATCGCAATCT-MGB*
Rotavirus	*NSP3*	*F, ACCATCTWCACRTRACCCTCTATGAG*<br>*R, GGTCACATAACGCCCCTATAGC*<br>*P, FAM-AGTTAAAAGCTAACACTGTCAAA-MGB*
Camplobacter jejuni	*cadF*	*F, CWGCTAAACCATARAAATAAAATTTCTCAC*<br>*R, YTTTGAAGGTAATTTAGATATGGATAATCG*<br>*P, VIC-CATTTTGAYGATTTTTGGCTTGA-MGB*
	hipO	*F, CTTGCGGTCATGATGGACATAC*<br>*R, AGCACCACCCAAACCCTCTTCA*<br>*P, FAM-TGCTTGCTGCAAAGTATT-MGB*
Camplobacter coli	*GlyA*	*F, AAACCAAAGCTTATCGTGTGC*<br>*R, AGTGCAGCAATGTGTGCAAT*<br>*P, FAM-TAAGCTCCAACTTCATCCG-MGB*
LT-ETEC	*LT*	*F, TTCCCACCGGATCACCAA*<br>*R, CAACCTTGTGGTGCATGATGA*<br>*P, FAM-CTTGGAGAGAAGAACCCT-MGB*
ST-ETEC	*STh*	*F, GCTAAACCAGYAGRGTCTTCAAAA*<br>*R, CCCGGTACARGCAGGATTACAACA*<br>*P, FAM-TGGTCCTGAAAGCATGAA-MGB*
	STp	*F, TGAATCACTTGACTCTTCAAAA*<br>*R, GGCAGGATTACAACAAAGTT*<br>*P, FAM-TGAACAACACATTTTACTGCT-MGB*
STEC	stx1	*F, ACTTCTCGACTGCAAAGACGTATG*<br>*R, ACAAATTATCCCCTGWGCCACTATC*<br>*P, FAM-CTCTGCAATAGGTACTCCA-MGB*
	stx2	*F, CCACATCGGTGTCTGTTATTAACC*<br>*R, GGTCAAAACGCGCCTGATAG*<br>*P, FAM-TTGCTGTGGATATACGAGG-MGB*
Shigella spp.	*ipaH*	*F, CCTTTTCCGCGTTCCTTGA*

		R, CGGAATCCGGAGGTATTGC<br>*P, VIC-CGCCTTTCCGATACCGTCTCTGCA-MGB*
Salmonella enterica	*ttr*	*F, CTCACCAGGAGATTACAACATGG*<br>*R, AGCTCAGACCAAAAGTGACCATC*<br>*P, FAM-CACCGACGGCGAGACCGACTTT-MGB*
Salmonella enterica serovar Typhi	*tviB*	*F, TGTGGTAAAGGAACTCGGTAAA*<br>*R, GACTTCCGATACCGGGATAATG*<br>*P, VIC-TGGATGCCGAAGAGGTAAGACGAGA-MGB*
	sty0201	*F, CGCGAAGTCAGAGTCGACATAG*<br>*R, AAGACCTCAACGCCGATCAC*<br>*P, FAM-CAGCCTGCTCCAGAACA-MGB*
Cryptosporidium hominus	*LIB13*	*F, TCCTTGAAATGAATATTTGTGACTCG*<br>*R, AAATGTGGTAGTTGCGGTTGAAA*<br>*P, FAM-CTTACTTCGTGGCGGCGT-MGB*
Cryptosporidium parvum	*LIB13*	*F, TCCTTGAAATGAATATTTGTGACTCG*<br>*R, TTAATGTGGTAGTTGCGGTTGAAC*<br>*P, FAM-TATCTCTTCGTAGCGGCGTA-MGB*
Cryptosporidium spp.	*18S*	*F, GGGTTGTATTTATTAGATAAAGAACCA*<br>*R, AGGCCAATACCCTACCGTCT*<br>*P, FAM-TGACATATCATTCAAGTTTCTGAC-MGB*
Entamoeba histolytica	*18S*	*F, ATTGTCGTGGCATCCTAACTCA*<br>*R, GCGGACGGCTCATTATAACA*<br>*P, FAM-TCATTGAATGAATTGGCCATTT-MGB*
Giardia spp.	*18S*	*F, GACGGCTCAGGACAACGGTT*<br>*R, TTGCCAGCGGTGTCCG*<br>*P, FAM-CCCGCGGCGGTCCCTGCTAG-MGB*

Supplemental Table 3: Infection prevalence for 14 enteric pathogen targets measured by Luminex xTAG Gastrointestinal Panel (GPP) and TaqMan Array Card (TAC) assays. Stool samples were tested from children at ages 6, 12, and 18 months old in Esmeraldas Province, Ecuador, 2022-2023. Created with script: : https://osf.io/ju8tw .

GPP Target	N	GPP pos	GPP Prev (95% CI)	TAC pos	TAC Prev (95% CI)
**Viruses**					
Adenovirus_40_41	154	14	9.1 (5.1, 14.8)	13	8.4 (4.6, 14.0)
Norovirus_GI	154	4	2.6 (0.7, 6.5)	7	4.5 (1.8, 9.1)
Norovirus_GII	154	10	6.5 (3.2, 11.6)	15	9.7 (5.6, 15.6)
Rotavirus_A	154	1	0.6 (0.0, 3.6)	12	7.8 (4.1, 13.2)
**Bacteria**					
Campylobacter	154	30	19.5 (13.5, 26.6)	44	28.6 (21.6, 36.4)
ETEC_LT	154	58	37.7 (30.0, 45.8)	57	37.0 (29.4, 45.2)
ETEC_ST	154	9	5.8 (2.7, 10.8)	23	14.9 (9.7, 21.6)
STEC_stx1	154	19	12.3 (7.6, 18.6)	15	9.7 (5.6, 15.6)
STEC_stx2	154	11	7.1 (3.6, 12.4)	6	3.9 (1.4, 8.3)
Shigella	154	25	16.2 (10.8, 23.0)	19	12.3 (7.6, 18.6)
Salmonella	154	124	80.5 (73.4, 86.5)	12	7.8 (4.1, 13.2)
**Protozoa**					
Cryptosporidium	154	17	11.0 (6.6, 17.1)	13	8.4 (4.6, 14.0)
Entamoeba_histolytica	154	2	1.3 (0.2, 4.6)	1	0.6 (0.0, 3.6)
Giardia	154	24	15.6 (10.2, 22.3)	31	20.1 (14.1, 27.3)

Supplemental Figure 1: Median fluorescence intensity (MFI) values for pathogen targets detected by the Luminex GPP Assay. Results are categorized according to TaqMan Array Card (TAC) and Luminex xTAG Gastrointestinal Pathogen Panel (GPP) assay sample results, for positive (+) and negative (–) detection for each target. Created with script: https://osf.io/jd5xz .

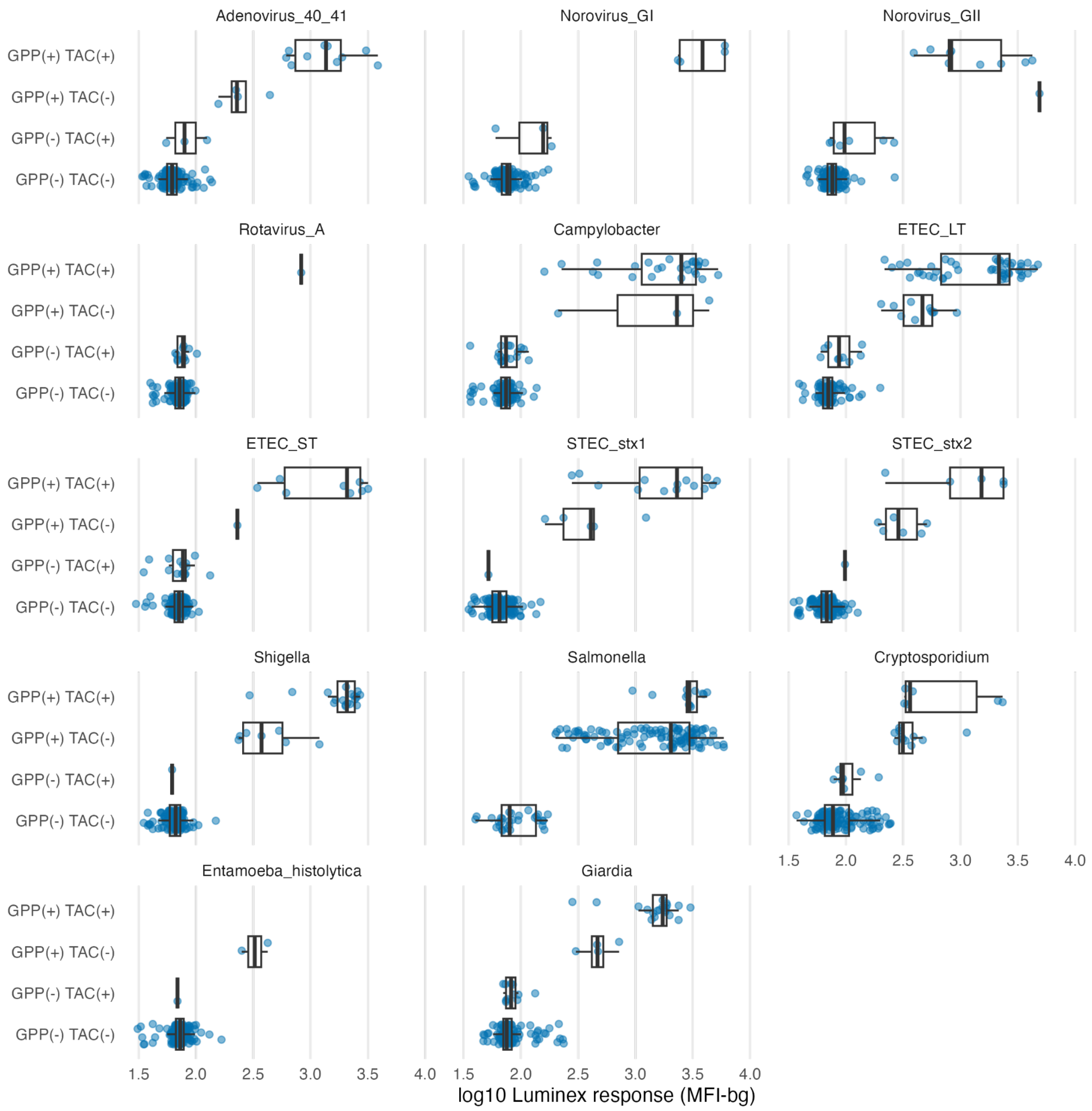

Supplemental Figure 2: Cycle threshold (Ct) values for pathogen associated gene targets detected by the TAC Assay. Multiple gene targets were used for some enteric pathogens in the TaqMan Array Card (TAC) panel. In each comparison, the top row label identifies the Luminex xTAG Gastrointestinal Pathogen Panel (GPP) target, and the second row identifies the TAC target. Results are categorized according to TAC and GPP assay sample results, for positive (+) and negative (–) detection for each GPP target. Created with script: https://osf.io/jd5xz .

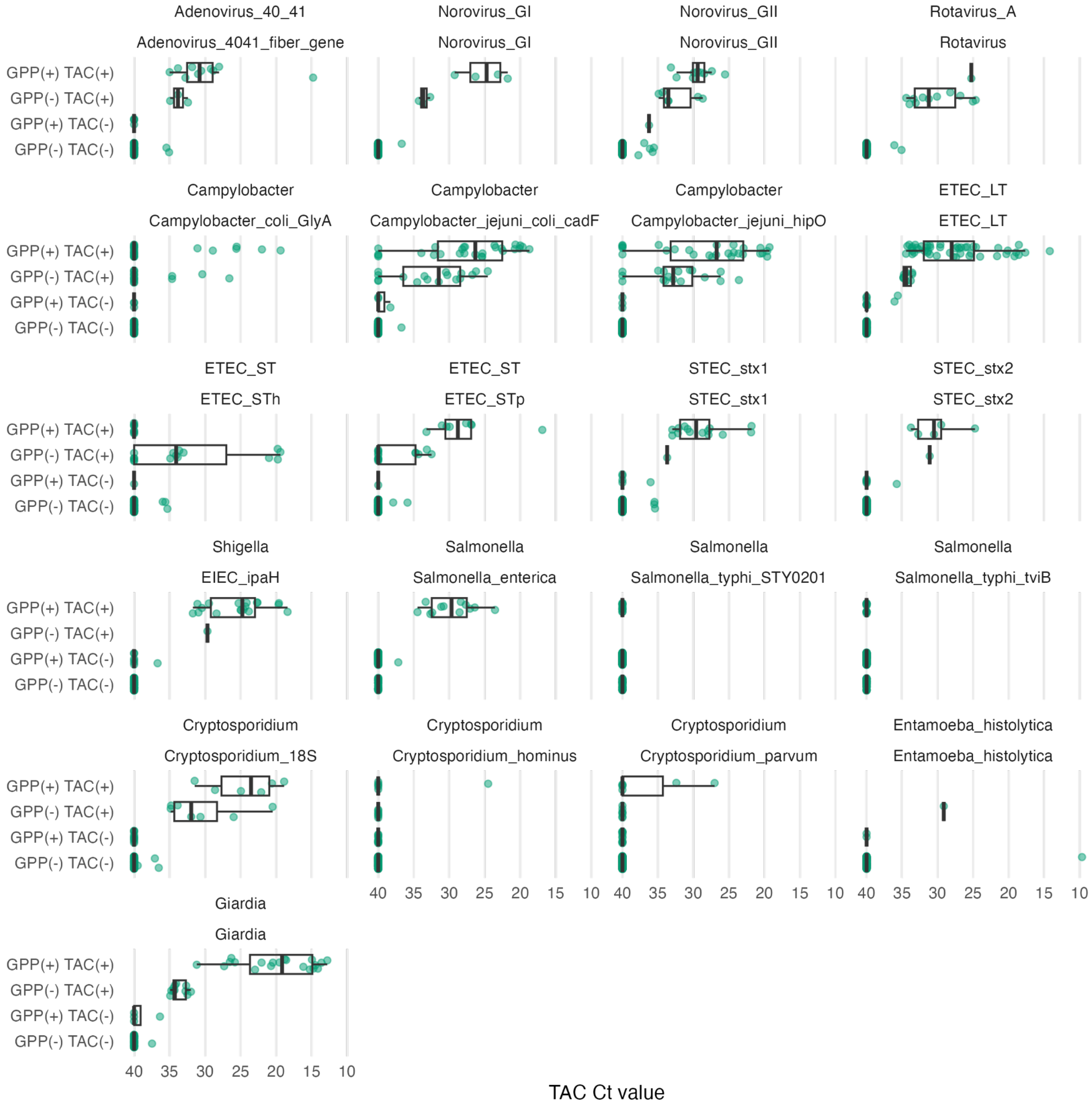

Supplemental Figure 3: Number of pathogens detected in each sample by assay. The number of pathogens detected by TaqMan Array Card (TAC) panel and the Luminex xTAG Gastrointestinal Pathogen Panel (GPP) exclude *Salmonella* due to the large number of apparent false positives identified by the GPP assay (main text, Figure 1). The number of pathogens detected could range from 0 to 10. Although distributions have the same median (1) and IQR (1, 2), the distributions differ slightly, with a slight shift toward more pathogens detected by the TAC assay (Wilcoxon signed rank $P = 0.014$) Note that 23 samples were negative to all TAC targets included in the analysis despite the sampling design, which conditioned on samples positive by TAC to at least one target on the GPP assay because we incorrectly included the EPEC-EHEC eae gene in sample selection (an error discovered after GPP testing).
Created with script: https://osf.io/v7cm6 .

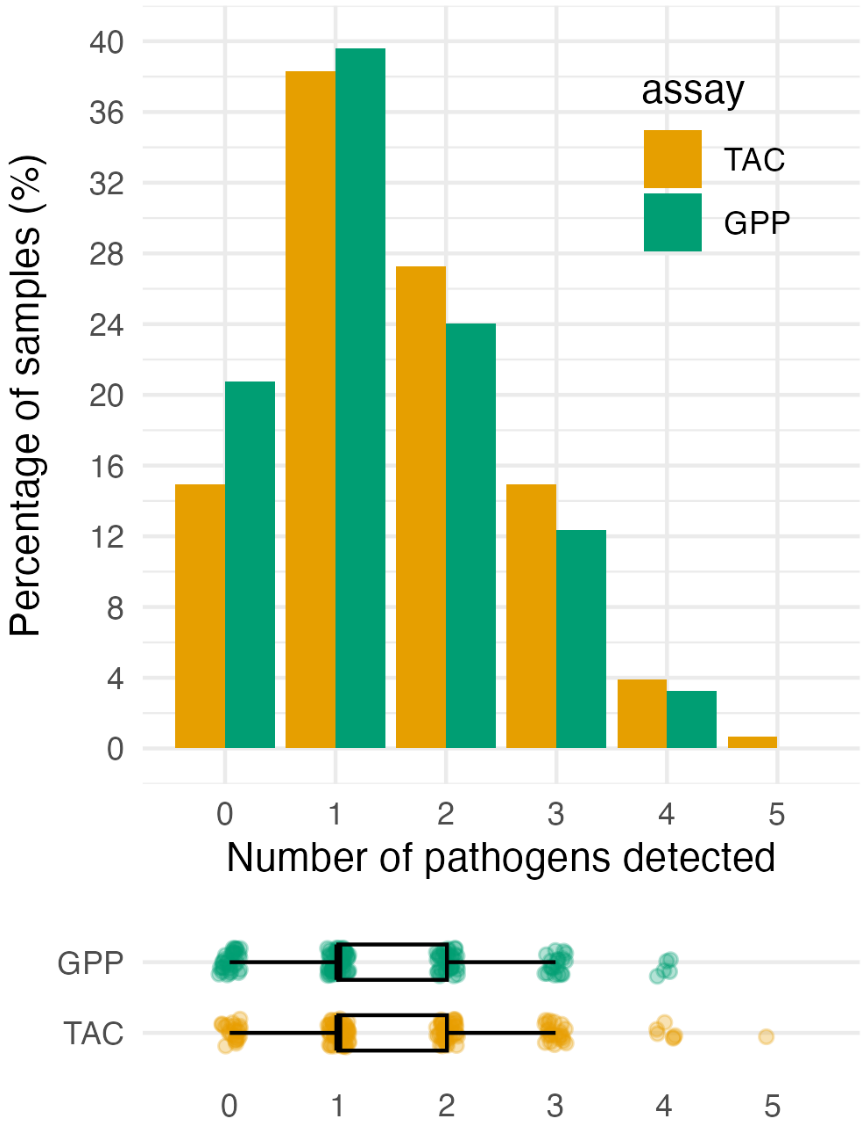

Supplement: ofaf027_Supplementary_Data [file ofaf027_supplementary_data.pdf]
